# Older African Americans' Experience of the Opioid Crisis

**DOI:** 10.1093/geroni/igaa057.261

**Published:** 2020-12-16

**Authors:** Sharon Rainer

**Affiliations:** Thomas Jefferson University, Moorestown, New Jersey, United States

## Abstract

In 2017, over 1200 Philadelphians died from opioid overdoses and an estimated 50,000 -70,000 people in the city are addicted to opioids. The opioid crisis is a family crisis that touches all communities affecting those using opioids, their family members, and the community. As policymakers develop responses to the opioid crisis, a multigenerational perspective is critical. With much published on the opioid crisis, there remains little understanding of older adult’s perceptions or experiences to this public health emergency. Older adults bring a unique voice. They are grandparents, parents, opioid users, and people living with chronic pain and/or addictions. Center in the Park (CIP), a nationally accredited senior center in Philadelphia, Pennsylvania sought to understand older African Americans’ perceptions of the opioid crisis and how their experiences inform senior center programming. CIP leadership sought a community partnership with Thomas Jefferson University College of Nursing to respond to what some in their community called “a domino effect” of the opioid crisis. Using a Community-Based Participatory Research Model rooted in humanistic research theory, a qualitative study was designed. Three focus groups (n29) were convened. All focus group recordings were transcribed and checked for accuracy. An iterative axial coding process was used. All transcripts were coded using the qualitative software program NVivo12. Findings suggest older adults are concerned about younger generations and addictions. In addition, older adults lack knowledge of the use and safety of opioids and have a general mistrust of the medical community to adequately limit the opioid epidemic.

